# Development of an intervention to reduce antibiotic use for childhood coughs in UK primary care using critical synthesis of multi-method research

**DOI:** 10.1186/s12874-017-0455-9

**Published:** 2017-12-28

**Authors:** Patricia J. Lucas, Jenny Ingram, Niamh M. Redmond, Christie Cabral, Sophie L. Turnbull, Alastair D. Hay

**Affiliations:** 1 School for Policy Studies, University of Bristol, 8 Priory Rd, Bristol, UK; 2 Centre for Child and Adolescent Health, Population Health Sciences, Bristol Medical School, University of Bristol, Bristol, UK; 3 National Institute for Health Research Collaborations for Leadership in Applied Health Research and Care West (NIHR CLAHRC West), University Hospitals Bristol NHS Foundation Trust, Bristol, UK; 4 Centre for Academic Primary Care, Population Health Sciences, Bristol Medical School, University of Bristol, Bristol, UK

**Keywords:** Intervention development, Respiratory tract infections, Child, Drug resistance, Microbial, Models, Psychological, Primary health care, Mixed methods, Evidence synthesis

## Abstract

**Background:**

Overuse of antibiotics contributes to the global threat of antimicrobial resistance. Antibiotic stewardship interventions address this threat by reducing the use of antibiotics in occasions or doses unlikely to be effective. We aimed to develop an evidence-based, theory-informed, intervention to reduce antibiotic prescriptions in primary care for childhood respiratory tract infections (RTI). This paper describes our methods for doing so.

**Methods:**

Green and Krueter’s Precede/Proceed logic model was used as a framework to integrate findings from a programme of research including 5 systematic reviews, 3 qualitative studies, and 1 cohort study. The model was populated using a strength of evidence approach, and developed with input from stakeholders including clinicians and parents.

**Results:**

The synthesis produced a series of evidence-based statements summarizing the quantitative and qualitative evidence for intervention elements most likely to result in changes in clinician behaviour. Current evidence suggests that interventions which reduce clinical uncertainty, reduce clinician/parent miscommunication, elicit parent concerns, make clear delayed or no-antibiotic recommendations, and provide clinicians with alternate treatment actions have the best chance of success. We designed a web-based within-consultation intervention to reduce clinician uncertainty and pressure to prescribe, designed to be used when children with RTI present to a prescribing clinician in primary care.

**Conclusions:**

We provide a worked example of methods for the development of future complex interventions in primary care, where multiple factors act on multiple actors within a complex system. Our synthesis provided intervention guidance, recommendations for practice, and highlighted evidence gaps, but questions remain about how best to implement these recommendations. The funding structure which enabled a single team of researchers to work on a multi-method programme of related studies (NIHR Programme Grant scheme) was key in our success.

**Trial registration:**

The feasibility study accompanying this intervention was prospectively registered with the ISRCTN registry (ISRCTN23547970), on 27 June 2014.

**Electronic supplementary material:**

The online version of this article (10.1186/s12874-017-0455-9) contains supplementary material, which is available to authorized users.

## Background

Antimicrobial resistance (AMR) is recognized as a global threat to human health, livelihoods and the economy [1–3]. Per annum 23,000 deaths in the USA, and 30,000 in the EU, are caused by treatment resistant bacteria [2]. In response to this threat, urgent policy action at national and international levels is needed including the development of AMR stewardships or responsible use strategies [2–5]. AMR stewardship encompasses strategies to ensure appropriate antimicrobial prescribing, including better targeting of treatment and reduced rates of prescription for self-limiting and non-bacterial infections [2, 6].

Current evidence suggests stewardship interventions in primary care can be effective in reducing antibiotic prescription and consumption rates [6, 7]. However, rigorous studies of carefully designed stewardship interventions, which recognize the complex multi-factorial social and behavioural influences on prescribing practice, monitor health impact, and include cost information are lacking [8]. The design of interventions targeting treatment of children is particularly challenging, given the multiple actors (clinician, child and parent) and behavioural triggers operating [9–13]. Childhood cough is the most common reason to attend primary care in the UK [8, 14], so identifying strategies to reducing antibiotics prescribed for respiratory tract infections (RTI) in childhood is important.

While guidance recommends an iterative approach to developing complex interventions [15], methods for achieving this are still emerging [16, 17] and intervention content and development are seldom presented in sufficient detail [18]. There is a need for greater clarity about the methods for designing interventions to change health professionals’ behaviour in particular [19]. As part of a 5-year programme of research we undertook linked primary research and systematic reviews [20–32]. The final study in this programme was to develop and pilot an intervention, synthesising across the prior studies to produce intervention recommendations informed by both theory and research [33, 34]. The purpose of this paper is to describe the methods of synthesis and intervention development, present our recommendations for stewardship strategies for clinical and research audiences, and describe our intervention in sufficient detail to allow replication (Additional file 1 TIDieR checklist) [18]. A feasibility study exploring the acceptability of the intervention and feasibility of a future effectiveness study is published elsewhere [35, 36].

## Methods

### The Programme of research

**Fig. 1:**
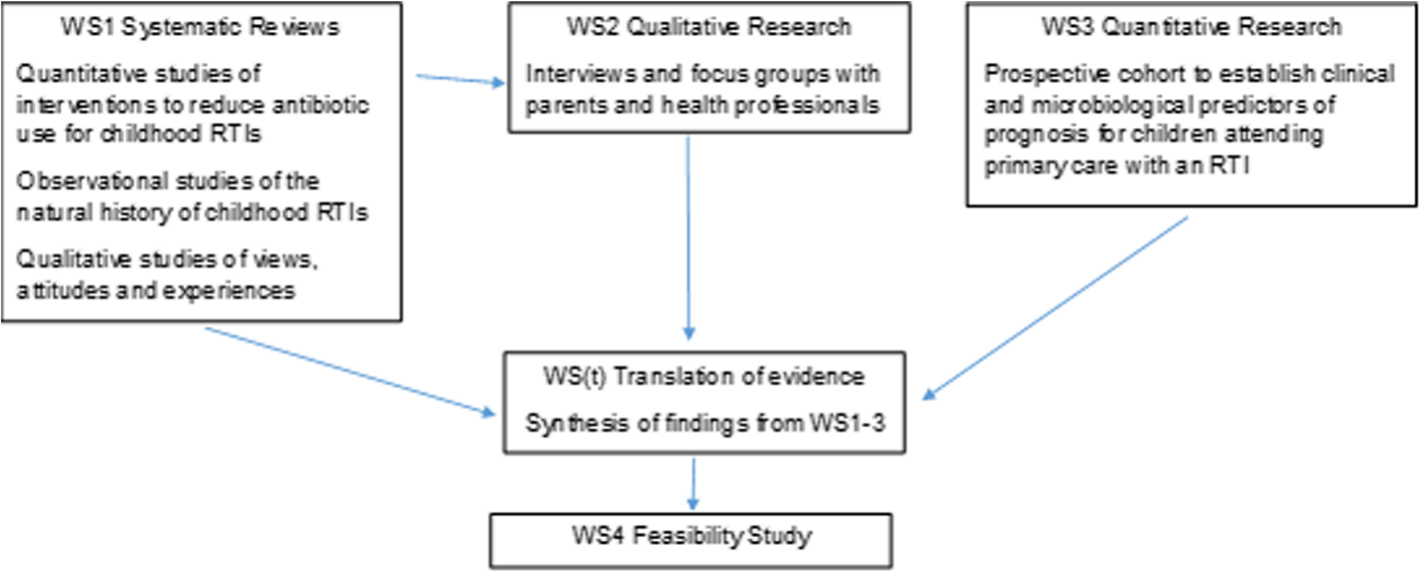
TARGET Schema

This paper reports on one of five workstreams (WS) in the TARGET programme of research. The research was conducted between 2010 and 2016, with the aim of improving the management of children presenting to primary care with cough and RTIs. Figure 1 provides a schematic diagram of the programme, and illustrates the sequential, multi-method programme of research. The methods for individual studies are reported elsewhere, appropriate ethical review was gained for all elements [20–28, 31, 36, 37]. Parent and clinician advisory groups contributed to all stages of the research. This paper reports on the translation WS, which synthesised findings from WS1–3 and translated these into a model for a novel intervention to be tested in a feasibility study (WS4) [35, 36].

Our model was therefore informed by:

1. Systematic review (SR) and meta-analysis of randomised controlled trials (RCTs) to reduce antibiotic prescribing for childhood RTIs [20].
2. SR of RCTs to alter parents’ consulting or antibiotic use when their children had an RTI [24].
3. SR with pooled symptoms estimates of observational studies charting the natural history of childhood RTIs [23].
4. SR and thematic synthesis of qualitative studies of the views, attitudes and experience of parents and clinicians about prescribing for minor childhood infections [38].
5. SR and meta-ethnography of observational qualitative studies of communication within consultations for acute childhood illness [25].
6. Interviews with parents exploring their experiences of consulting for childhood RTIs [39].
7. Focus groups and interviews establishing parents’ information needs and self-efficacy beliefs in relation to coughs [40].
8. Interviews conducted with health professionals explored perceptions about their own prescribing decisions [31].
9. Critical synthesis of qualitative research generating a new theoretical understanding of safety seeking in childhood consultations [27].
10. Large cohort study (>8300 children) presenting with acute cough RTI into a prospective observational study tracking hospitalization for RTI illness and children’s recovery in the 4 weeks following consultation providing a clinical prediction rule (CPR) for children at risk of hospitalization following RTI consultation [22, 32].

### Logic model selection

**Fig. 2:**
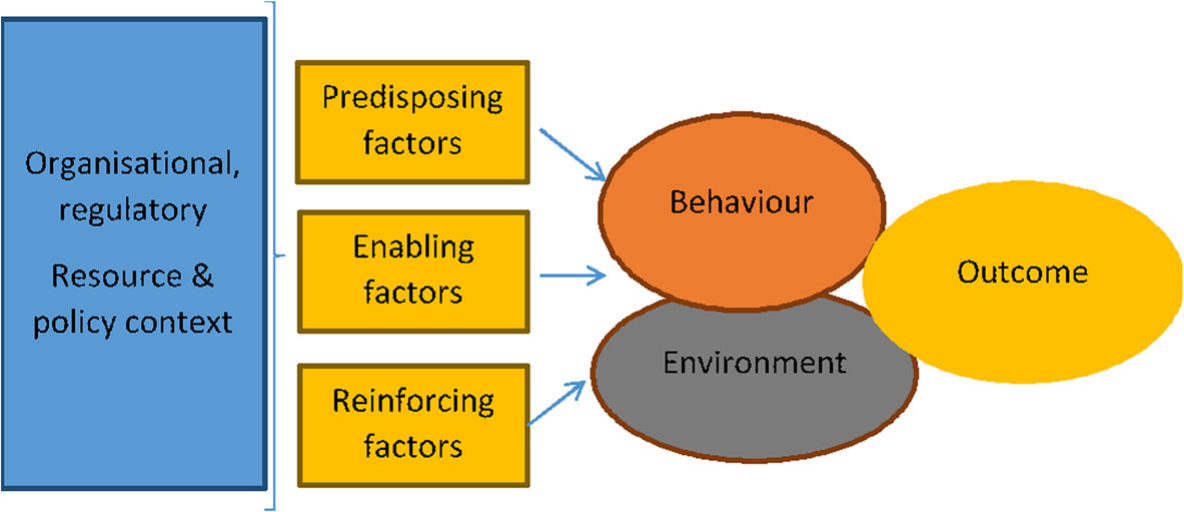
Simplified model of change, adapted from Green & Kreuter Precede/Proceed model of health promotion planning: http://lgreen.net/precede.htm

We identified Greene and Kreuter’s Precede/Proceed [41, 42] model as a vehicle for translating our findings into behavioural recommendations (Fig. 2). This model draws on social cognitive theories which hypothesize that behaviour is influenced by context and by personal perceptions of costs, benefits and efficacy of actions [43–45]. Social cognitive theories are frequently used to understand health behaviours, including in the development of logic models informing behavioural interventions [46–54]. The Precede/Proceed approach integrates these elements into a unified model describing the interaction between the physical and social environment and individuals’ motivations and intentions within a given health system [52]. Intervention planning (the ‘precede’ phase) is achieved by describing the circumstances under which a desired outcome comes about. The determinants of the outcome are divided into predisposing, enabling and reinforcing factors. Planning progresses to the ‘proceed’ phase by considering how change might be brought about and evaluated. This model has been used for health promotion and corresponding evaluations across a range of health topics, including understanding the use of antibiotics for minor child illness [55] and to synthesize qualitative and quantitative studies [56, 57].

Planning begins with defining the desired outcome, and we achieved this through discussion and consensus. The TARGET programme research team, parent and clinician advisory groups discussed potential outcomes of interest for policy makers, clinicians, parents and children. Outcomes considered included antibiotic prescription, antibiotic consumption, health experiences, and child health status. Our aim was to reduce antibiotic use without compromising health experience or status. In the UK, antibiotics cannot be accessed without a prescription from a qualified clinician, i.e. consumption is contingent upon prescription. We therefore chose antibiotic prescription as our target behaviour, and reduction in number of prescriptions to children with RTI as our primary outcome. We recorded hospitalisation for complication of RTI as an indicator of a poor health outcome.

### Synthesis of findings

The behavioural target of this intervention was clinicians’ prescribing decisions. As a team, we reviewed all the findings from the SRs and primary research conducted in TARGET to identify drivers of the decision to prescribe. The team of researchers responsible for each study element produced a summary of key findings which commented on contexts or behaviours which predisposed, reinforced, or enabled the antibiotic prescribing decision for children with a RTI presenting in primary care. The synthesis team (PJL, JI, NR) assessed the weight of evidence for all factors identified. Where evidence was drawn from multiple sources we assigned a stronger evidence rating; for example, from several papers in systematic reviews, or from more than one study source. These evidence statements were presented to the full programme team and to the expert advisory group for discussion, comment and agreement.

### Intervention development and feasibility testing

We used the evidence statements to develop intervention elements, in consultation with our programme team, programme steering committee, and parent and clinician advisory groups. A feasibility study established the acceptability and usability of our intervention [35, 36].

## Results

**Fig. 3:**
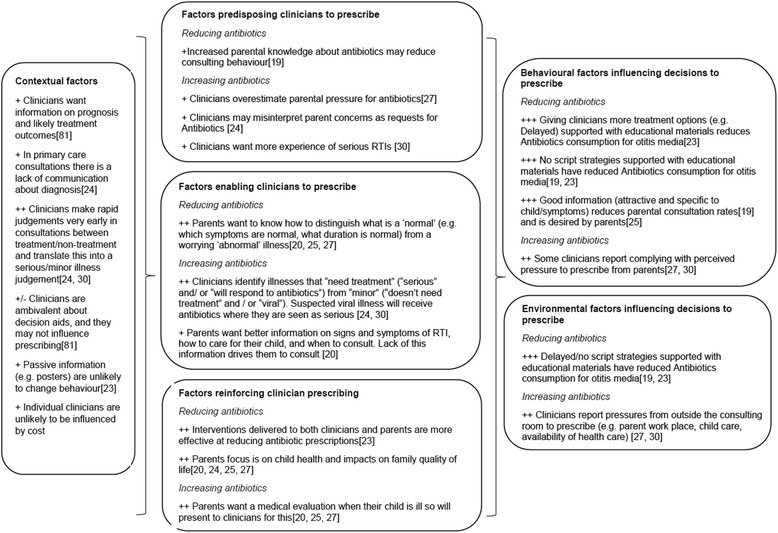
Summary Evidence Statements fitted to model

The findings produced within the TARGET programme (including systematic reviews of existing evidence and primary research) provide the data for this study. The results of the evidence mapping and synthesis exercise are shown in Fig. 3, where we have mapped the evidence statements to the model components (Fig. 2). The number of ‘+’ signs indicates the strength of evidence supporting each statement, where a single + indicates evidence from only one source, and three ‘+‘s evidence from multiple sources including systematic reviews. Citations to published findings are provided, but the synthesis included unpublished findings at the time.

Organisational and policy level changes (e.g. the introduction of prescribing guidelines, financial consequences, or changes to access to antibiotics) were not tested in the literature we reviewed but would be consistent with the model. In our discussion of the model we also predicted that making antibiotic prescriptions more visible to self and peers (ie systems for monitoring and peer sanctions) were plausible routes to discourage prescribing, but again these were not tested in the literature we reviewed and not mentioned by our participants.

### Intervention recommendations

Evidence from past intervention studies clearly suggested that passive information for either parents or clinicians was unlikely to change antibiotic prescribing behaviour, but providing alternative treatment options probably would. Our analysis identified drivers of prescribing underexplored in the literature: the role of clinician uncertainty about diagnosis and prognosis, and the implications of miscommunications between parents and clinicians during consultation. The latter refers not to generic communication skills, but specific miscommunications about parents’ desires and concerns, and clinicians’ no-treatment communications.

We used the evidence statements (Fig. 3) to develop a series of intervention recommendations describing the features of interventions delivered to individuals or groups considered most likely to be effective in changing antibiotic prescribing (Table 1, ordered from the strongest to the weakest evidence).

**Table 1:** Recommendations for interventions to change clinician prescribing behavior

An intervention to change clinician prescribing behavior SHOULD:	An intervention to change clinician prescribing behavior SHOULD NOT:
1. Give explicit antibiotic prescription recommendations 2. Give alternative treatment options (including for parents e.g. home care advice, and clinicians e.g. delayed scripts) 3. Address the treatment/no treatment distinction made by clinicians 4. Give information on specific symptoms 5. Should address both clinicians AND parents (though we don’t know if there is a difference if they receive information together) 6. Should provide information on prognosis that is tailored to the child and addresses the common and/or stated (not implied) concerns of parents 7. Address known environmental pressures (e.g. external pressures to prescribe/consult) 8. Should make clinicians feel more confident/experienced 9. Acknowledge treatment decisions in care of childhood RTIs are usually made in the absence of definitive diagnosis. Novel methods to reduce uncertainty may be helpful. 10. Be designed in consultation with clinicians and parents	1. Work against the environment in which clinician operates (e.g. in conflict with targets) 2. Be generic 3. Patronize or undermine parental or clinician decision making 4. Be passive (e.g. posters) 5. Increase anxiety or perception of risk for either party

**Fig. 4:**
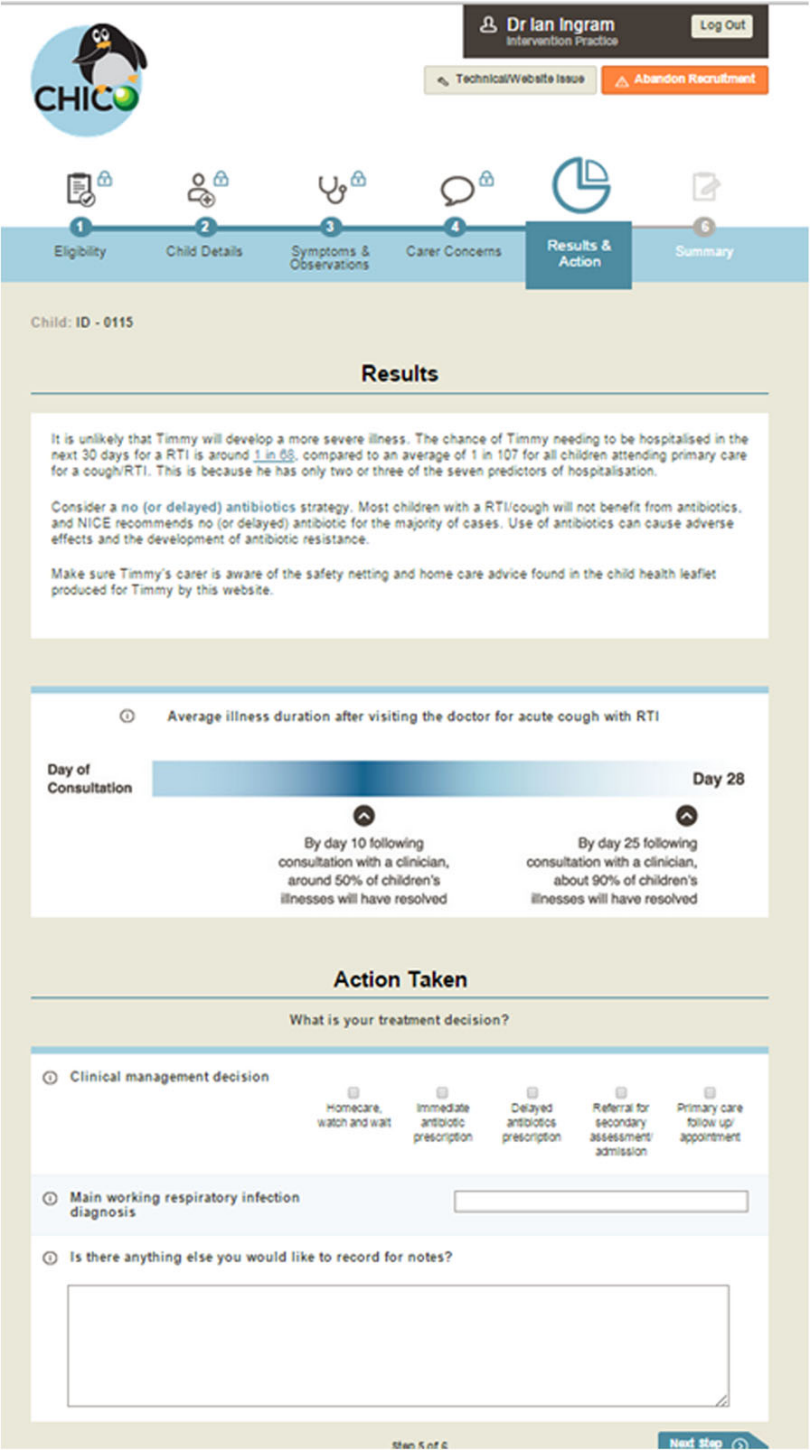
Example Clinical Prediction Rule and Prescription Guidance Results Screen

We argued that the intervention developed should aim to decrease clinical uncertainty about prognosis, provide clinicians with an action to support no-antibiotic treatment strategies, and reduce miscommunication about parent concerns. Given the need to respond to consultation content (CPR and parent concerns) we chose to develop an interactive web-based intervention accessible from clinicians’ desktops. While the intervention aims were evidence-based, we struggled to find literature to guide intervention design elements such as layout, look and personalisation of information. We worked with a graphic designer and web-development agency to ensure the intervention was attractive, easy to navigate, and personalized to each child. The intervention elements were tested with parents and clinicians using example materials and wireframes (showing the functional elements of the website). Wording was taken from NICE advice [58] supplemented with TARGET findings [23, 30] to ensure information provided was in line with best current evidence. We worked with a commercial web-design agency to develop the web-based tool, and a graphic designer to provide attractive images and a logo for our study (see Fig. 4). A functional prototype was tested by general practitioners to test clarity of instructions and to improve usability.

### Intervention process

Clinicians were asked to open a web-page when a child presented with a RTI, and login to a website which provided both study information and the intervention. The intervention comprised three active elements: explicit elicitation of parent concerns and expectations (to reduced clinician-perceived pressure to prescribe), the results of a CPR accompanied by delayed or no-antibiotic guidance (to reduce clinical uncertainty), and provision of a personalized printout for carers (to provide an alternate treatment action for clinicians).

**Fig. 5:**
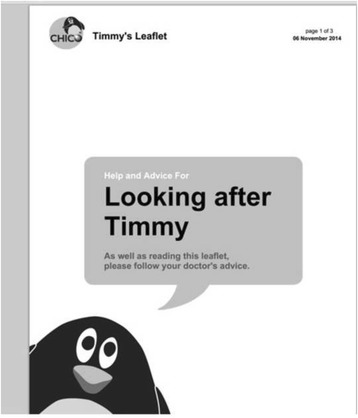
Example of Individualized Parent-facing Leaflet

Clinicians in the treatment arm were guided through 5 webpages. Pages 1 and 2 required recording of sociodemographic and eligibility criteria, clinical observations and parent reported symptoms (forced questions) which drove the CPR. On page 3 clinicians were asked “Please ask the parent/carer what their worries and expectations are today. Is there anything in particular they wanted to talk about?” and provided with a list of common concerns plus a free text box. If no concerns were recorded an alert appeared to confirm the parent/carer had no concerns. Page 4 provided the results of the CPR as numerical relative risk of hospitalization along with antibiotic treatment recommendations, and information about likely illness duration (Fig. 4). Page 4 requested clinicians select their treatment decision from homecare, immediate antibiotic, delayed antibiotic, referral to secondary care, primary care follow-up appointment (multiple selections permitted). Selection of their treatment option(s), together with clinical signs, symptoms and parent concerns generated a parent-facing leaflet (Fig. 5) which we presented as a print option on page 5. This leaflet was individualized with the child and clinician’s name, date, home care advice responding to symptoms, concerns and treatment selected, and standard safety-netting advice (Additional file 2).

### Testing feasibility

The results of the feasibility study are published elsewhere [35]. We found the intervention components themselves acceptable and the parent leaflet highly appreciated by some clinicians. However, the use of a stand-alone website that required login was a barrier to use.

## Discussion

### Summary

We successfully used the Precede/Proceed to structure a synthesis of the findings from a programme of study to inform a complex intervention. We concluded that interventions which reduce clinical uncertainty, reduce clinician/parent miscommunication, make clear delayed/no-antibiotic recommendations, and provide clinicians with alternate treatment actions have the best chance of reducing antibiotic prescriptions in primary care for childhood RTI.

Our synthesis provides a method for the development of future complex interventions in primary care using a theoretical framework combined with empirical findings. This publication makes explicit the steps we undertook in intervention design prior to piloting, and enables examination of any changes we might make to the intervention design between feasibility and effectiveness studies [19].

### Strengths and weaknesses of our approach

The strength of our approach was to allow strength of evidence to be considered within theoretically driven logic model development. We integrated quantitative and qualitative findings to develop an evidence-based model that reflected the experiences and views of parents and clinicians. We produced generalizable intervention recommendations, as well as the intervention developed for this context. This was possible because of the context of a National Institute for Health Research programme grant, enabling the same group of researchers to work together across phases of intervention development [15], and working in an interdisciplinary team in a multi-method environment. We therefore see our funding context are a key element in our success.

The weakness of this approach was in moving from intervention recommendations, to decisions about how to implement these in practice. While we were confident about the principles guiding intervention development, we had to rely on expert opinion and traditional testing with users to inform the many practical decisions involved in designing the intervention. The effectiveness of web-based interventions varies greatly [59] and very little is known at present about the mechanisms through which interventions work. Limited systematic research had been conducted to identify the design features most likely to result in behavioural change [60, 61].

An advantage of the Precede/Proceed framework includes consideration of actions to change social norms, regulatory constraints, resource availability. However, our application of it was limited by the research evidence available concerning childhood RTIs. Within both the primary data we generated and the systematic reviews we undertook in our TARGET programme, there was a focus on drivers of prescribing within the patient consultation. The decision aids and clinician communication tools that act within consultations sit within a broader range of interventions which act to change the context for consultations. Recent evidence for population-level changes suggests that shifting social norms through mass communication [62] and prescriber feedback [63] can influence antibiotic prescribing behaviour.

We underestimated the barriers to use of a stand-alone website, and were not able to integrate this intervention into existing electronic medical notes systems.

### Comparison to similar literature

Interventions informed by evidence *and* theory have the best chance of altering health related behaviours [15]. However, methods and advice for how to achieve this to develop a specific intervention are sparse [17, 64, 65]. We believe our combination of strength of evidence applied to a theoretical structure, augmented with input from key stakeholders provides a useful model to achieve this.

We drew on a developing literature using logic models to inform mixed methods syntheses of research [66, 67]. Our work complements the contemporaneous work of Yardley and colleagues in developing their ‘person-based approach to intervention development’ which similarly combines mixed methods primary research with theoretical models [68]. Michie’s behaviour change wheel usefully synthesised behaviour change theories to identify key influences on individual behaviours [69]. Our strength of evidence approach adds to this body of work a mechanism for identifying which actions were most likely to elicit change in this case.

Our approach is similar to those employed by O’Brien and colleagues to develop a web-based healthy lifestyles intervention for older adults [65] and Salisbury and colleagues to develop a new telehealth intervention for chronic disease in adults using Precede/Proceed [57]. Both of these studies used systematic reviews of the existing evidence, supplemented with additional primary research and stakeholder consultation [57], or co-design workshops [65]. Both study teams describe the success of this structured approached, and the tele-health intervention has since been tested, with modest benefits for patient health [70]. However, like us, while the evidence review provided functional guidance, authors report little guidance about the design features of the website [65].

Responding to the scale of the AMR challenge, there are very many interventions aimed at reducing antibiotic prescribing for RTIs in primary care, although fewer focus on children specifically. Many combine approaches drawing on previous research to suggest intervention elements. Four recent trials of family practitioner training using previous research to establish effective interventions and combine approaches in a new multi-component intervention with mixed success [63, 71–73], in one case highlighting differential effects by age where a reduction in prescriptions was seen for adults and adolescents, but not children under 12 [71]. Two studies are ongoing [74, 75]. These approaches to changing the environment for prescribing and use of social norms including through peers are consistent with our model.

There is a growing literature testing methods to change professionals’ behaviour. Prior literature establishes that education alone is insufficient to change prescribing behaviour in primary care [76], although we know that computerised reminders can be effective [77]. Our research contributes to the gap identified in using computerised reminders as part of a more complex decision support system [77], but our intervention would benefit from better integration into electronic health record systems.

## Conclusions

### Implications for research

Using the Precede/Proceed model successfully enabled the integration of multiple studies into a critical synthesis of evidence to inform development of a complex behaviour change intervention. The intervention benefitted from the inclusion of qualitative research, which provided rich data to support theory generation, interpretation of quantitative findings, and content for the intervention. Quantitative research provided the best available evidence on likely effective actions, and on the natural history of childhood coughs which are common, minor, and burdensome for children, families and health services. This method for developing a model of behavioural change also enabled the identification elements that would be useful in a future process evaluation.

Research into moving from intervention aims and behavioural targets, to advice for intervention design and delivery would be valuable [17, 65]. For example, while our aim to reduce clinical uncertainty was clear, how and when to present the CPR and symptom duration information to achieve this was a matter of opinion. Although we were guided by the views and experiences of clinicians in our team, steering group, and advisory groups, the challenge of presenting scientifically accurate data (with appropriate confidence boundaries) as a reassuring statement was considerable.

We moved outside the evidence we had gathered, and adopted best practice from other studies in producing tailored advice for each child [78]. One of the advantages of web-based health interventions is the increased facility for tailoring and presenting individualized information which may be more engaging, relevant and motivating [79]. While we know that clinicians liked this feature, we do not yet know which elements and in what circumstance such tailoring makes a difference.

Making explicit the methods for intervention development is an important step in improving transparency in intervention design and testing. This publication demonstrates one approach to this, and will allow examination of future changes to our intervention.

### Implications for practice

Our model suggests actions that can be taken by individual clinicians and medical practices to reduce their use of antibiotics. These include creating an environment where non-use of antibiotics is viewed positively, including considering how to stop ‘no-treatment’ decisions feeling like a non-action decision for clinicians; ensuring parents know how to recognise ‘abnormal’ symptoms and (conversely) what is expected when a child is ill; and discouraging clinicians from using the language of serious/minor illness to describe treatable/non-treatable cases. These actions should reduce pressures to prescribe within consultations. We have developed one particular intervention responding to these within-consultation factors. But other solutions, including at population, regulatory and policy level, are consistent with our model and could be usefully tested in practice.

## Additional files

Additional file 1: TIDieR Checklist. (DOCX 29 kb)

Additional file 2: Responsive Safety Netting Advice. (DOCX 23 kb)

## Abbreviations

AMR: Antimicrobial resistance
CPR: Clinical prediction rule
RCT: randomised controlled trial
RTI: Respiratory tract infections
SR: Systematic review
WS: Workstream
