# Supplementary material for: Development of an intervention to reduce antibiotic use for childhood coughs in UK primary care using critical synthesis of multi-method research
Source: BMC Med Res Methodol. 2017 Dec 28;17:175. doi: 10.1186/s12874-017-0455-9 (PMC5745782; doi:10.1186/s12874-017-0455-9)
Supplement: Supplementary file 1 — TIDieR Checklist. (DOCX 29 kb) [file 12874_2017_455_MOESM1_ESM.docx]

**
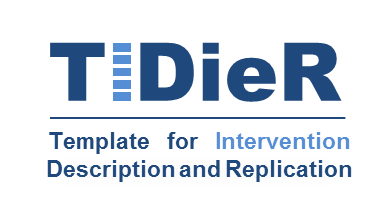
The TIDieR (Template for Intervention Description and Replication) Checklist*:**

Information to include when describing an intervention and the location of the information

| **Item number** | **Item** | **Where located **** | |
| --- | --- | --- | --- |
|  |  | Primary paper  (page or appendix  number) | Other ^†^ (details) |
|  | **BRIEF NAME** |  |  |
| **1.** | Provide the name or a phrase that describes the intervention. | __Abstract__ | ”a web-based within-consultation intervention to reduce clinician uncertainty and pressured to prescribe designed to be used when children with RTI present to a prescribing health professional in primary care” |
|  | **WHY** |  |  |
| **2.** | Describe any rationale, theory, or goal of the elements essential to the intervention. | Abstract, methods and Box 1 contents | We used Green & Kreuter’s Preced-Proceed model to formulate intervention recommendations which we list in Box 1 |
|  | **WHAT** |  |  |
| **3.** | Materials: Describe any physical or informational materials used in the intervention, including those provided to participants or used in intervention delivery or in training of intervention providers. Provide information on where the materials can be accessed (e.g. online appendix, URL). | Figures 3 & 4 | Example materials are provided |
| **4.** | Procedures: Describe each of the procedures, activities, and/or processes used in the intervention, including any enabling or support activities. | (add) | We cite the paper describing our prognostic algorithm in full |
|  | **WHO PROVIDED** |  |  |
| **5.** | For each category of intervention provider (e.g. psychologist, nursing assistant), describe their expertise, background and any specific training given. | Abstract and (add cite) | We describe these “prescribing health professionals in primary care” and describe the target sample in full in our accompanying protocol paper which we cite here. |
|  | **HOW** |  |  |
| **6.** | Describe the modes of delivery (e.g. face-to-face or by some other mechanism, such as internet or telephone) of the intervention and whether it was provided individually or in a group. | Abstract | Web-based |
|  | **WHERE** |  |  |
| **7.** | Describe the type(s) of location(s) where the intervention occurred, including any necessary infrastructure or relevant features. | Throughout and (add cite) | We describe the intervention as designed for primary care, and our accompanying protocol paper describes the target sample in full |
|  | **WHEN and HOW MUCH** |  |  |
| **8.** | Describe the number of times the intervention was delivered and over what period of time including the number of sessions, their schedule, and their duration, intensity or dose. | _(add cite)___ | _This paper describe intervention development, this information is provided in the forthcoming feasibility study paper. |
|  | **TAILORING** |  |  |
| **9.** | If the intervention was planned to be personalised, titrated or adapted, then describe what, why, when, and how. | _p.x_______ | “Selection of their treatment option, together with clinical signs and symptoms and recorded parent concerns generated a parent-facing leaflet which we presented as a print option on the final page. This leaflet included a summary of the treatment option selected, advice about home care responding to their symptoms and parent-reported concerns, and standard safety-netting advice. This leaflet was individualized with the child and clinician’s name, date, and details of the current illness” |
|  | **MODIFICATIONS** |  |  |
| **10.^ǂ^** | If the intervention was modified during the course of the study, describe the changes (what, why, when, and how). | _n/a_____ | Modifications to the intervention were not made in this stage of the study |
|  | **HOW WELL** |  |  |
| **11.** | Planned: If intervention adherence or fidelity was assessed, describe how and by whom, and if any strategies were used to maintain or improve fidelity, describe them. | _(add cite)___ | _This paper describe intervention development, this information is provided in the protocol paper cited here. |
| **12.^ǂ^** | Actual: If intervention adherence or fidelity was assessed, describe the extent to which the intervention was delivered as planned. | _(add cite)___ | _This paper describe intervention development, this information is provided in the forthcoming feasibility study paper. |

** **Authors** - use N/A if an item is not applicable for the intervention being described. **Reviewers** – use ‘?’ if information about the element is not reported/not sufficiently reported.

† If the information is not provided in the primary paper, give details of where this information is available. This may include locations such as a published protocol or other published papers (provide citation details) or a website (provide the URL).

ǂ If completing the TIDieR checklist for a protocol, these items are not relevant to the protocol and cannot be described until the study is complete.

* We strongly recommend using this checklist in conjunction with the TIDieR guide (see *BMJ* 2014;348:g1687) which contains an explanation and elaboration for each item.

* The focus of TIDieR is on reporting details of the intervention elements (and where relevant, comparison elements) of a study. Other elements and methodological features of studies are covered by other reporting statements and checklists and have not been duplicated as part of the TIDieR checklist. When a **randomised trial** is being reported, the TIDieR checklist should be used in conjunction with the CONSORT statement (see [www.consort-statement.org](http://www.consort-statement.org)) as an extension of **Item 5 of the CONSORT 2010 Statement.** When a **clinical trial** **protocol** is being reported, the TIDieR checklist should be used in conjunction with the SPIRIT statement as an extension of **Item 11 of the SPIRIT 2013 Statement** (see [www.spirit-statement.org](http://www.spirit-statement.org)). For alternate study designs, TIDieR can be used in conjunction with the appropriate checklist for that study design (see [www.equator-network.org](http://www.equator-network.org)).
