# Supplementary material for: Development of an intervention to reduce antibiotic use for childhood coughs in UK primary care using critical synthesis of multi-method research
Source: BMC Med Res Methodol. 2017 Dec 28;17:175. doi: 10.1186/s12874-017-0455-9 (PMC5745782; doi:10.1186/s12874-017-0455-9)
Supplement: Supplementary file 2 — Responsive Safety Netting Advice. (DOCX 23 kb) [file 12874_2017_455_MOESM2_ESM.docx]

**Web Page Title: What to look out for & when to see the Doctor urgently**

| Responsive rule | Text to show |
| --- | --- |
| First sentence for Children > 12 months age  DO NOT generate if immediate referral | Children’s illnesses often get better and worse over the day, so watching HIS/HER illness over time is useful. More severe illnesses will usually get worse and worse.  It is not possible to list every illness symptom that might concern a doctor, but we hope the following will help with **this** illness. Remember that dangerous complications are very rare in children without ongoing significant health problems.  Children with any of these signs should be seen by a doctor urgently (Usually the same day): |
| First sentence for Children < 12 months age  DO NOT generate if immediate referral | Children’s illnesses often get better and worse over the day, but babies can become more ill quite quickly. Since NAME is still young, it is particularly important to watch out for HIS/HER illness **getting worse and staying worse**.  It is not possible to list every illness symptom that might concern a doctor, but we hope the following will help with **this** illness. Remember that dangerous complications are very rare in children without ongoing significant health problems.  Children with any of these signs should be seen by a doctor urgently (Usually the same day): |
| First sentence for children referred for admission | **Your doctor has decided that NAME needs to be seen by a specialist at the hospital.**  The following are some signs of more severe illness that it is important for parents to know about: |
| Always | • If HIS/HER hands, feet or lips start turning bluish or much paler than usual, or if her hands or feet are very cold while her body is warm. |
| [Only show if between 3 & 6 months of age] | - If HE/SHE has a temperature of 39^o^ C or more |
| [only show if under 12 months] | - If HE/SHE STOPS feeding entirely |
| ALL | Children with any of these signs should be seen by a doctor straight away (either phone 999 for an ambulance or take them immediately to your nearest A&E department): |
| Always | - Serious breathing problems - including rapid breathing and being short of breath (as if NAME has been running) or is ‘working hard’ to breath (it might look as though the skin between the ribs and below the ribs get sucked in each time SHE/HE takes a breath). - If HIS/HER tongue begins to look blue - Cannot be woken or wakes only slightly and then immediately goes back to sleep. |
| If Asthma present | Make sure that you have discussed what to do when NAME has a cough or cold with your GP or Asthma nurse. NAME should have an asthma management plan that will give specific advice about what to do and what to look out for when HE/SHE is unwell. |
| All | Your emergency contact details are:  **GP Contact:**  Tel: [local number]  **GP Out of Hours Contact:**  Tel: [local number]  Accident and Emergency:  [local address] |
| All | You should always ask for more advice from a healthcare professional if you continue to be very worried. |
